# Incidence of Malignancy and Risk Factors Associated with Kidney Transplant Patients

**DOI:** 10.31661/gmj.v13i.3518

**Published:** 2024-10-21

**Authors:** Reza Marvi Jadidi, Azam Mivefroshan, Yousef Roosta

**Affiliations:** ^1^ Department of Internal Medicine, School of Medicine, Urmia University of Medical Sciences, Urmia, Iran

**Keywords:** Post-transplant Malignancy, Kidney Transplantation, Risk Factor, Incidence

## Abstract

Background: The incidence and risk factors associated with the development of post-transplant malignancies contributes to increased morbidity among kidney transplant recipients were examined in this study. Materials and Methods: A retrospective cross-sectional analysis was conducted to evaluate the medical records of all kidney transplant recipients at Urmia Imam Khomeini Hospital from January 2000 to December 2020. Patients were stratified into two groups based on the presence or absence of post-transplant malignancy. Demographic data, comorbidities, cancer history, and immunosuppression regimens were collected and compared between the groups. Statistical significance was determined using appropriate tests, including the t-test, Mann-Whitney test, Pearson chi-square test, and Fisher exact test. All analyses were performed using SPSS 21, and a P-value of less than 0.05 was considered statistically significant. Results: Of the 4070 kidney transplant recipients, 3042 (74.7%) were male and 1028 (25.3%) were female. The mean age at malignancy diagnosis was 53.78 years (standard deviation ± 14.24). The overall incidence of post-transplant malignancy was 9.6% per 1,000 patients (95% confidence interval: 9.6-13.2). Incidence rates varied significantly by age group: 4.6% for those younger than 30, 7.6% for those aged 30-50, and 29.3% for those older than 50 (P0.001). A small percentage of patients (n=3, 7.7%) required the addition of antithymocyte globulin (ATG) to their primary immunosuppression regimen. The most commonly used immunosuppressive regimens were prednisolone in combination with either sandimune and azathioprine or sandimune and cellcept, employed in 35.9% of patients. The most common underlying causes of kidney failure were glomerulonephritis (GN) and hypertension (HTN), accounting for 38.5% and 35.9% of cases, respectively. Conclusion: Kidney transplant recipients demonstrated a higher incidence of post-transplant malignancies. Male sex, older recipient age, and a history of underlying diseases were identified as significant risk factors for malignancy development. The primary cause of kidney failure among the patients was GN, followed by HTN.

## Introduction

Renal replacement therapy (RRT) is essential for patients with severe renal dysfunction. Kidney transplantation (KT) offers a well-established survival benefit and enhanced quality of life compared to dialysis [1][2]. Recent decades have seen substantial improvements in graft survival post-KT, primarily due to the advent of novel immunosuppressive agents such as cyclosporine, tacrolimus, and mycophenolate mofetil [3]. Moreover, reduced post-transplant cardiovascular mortality has contributed to improved patient survival following KT [4]. However, the ongoing challenge in post-transplant management lies in mitigating the heightened risk of malignancies following KT.

KT is considered the gold standard treatment for end-stage renal disease, providing superior survival and quality of life compared to dialysis [1][2]. However, post-transplant malignancies (PTM) remain a significant complication, representing the second leading cause of mortality among kidney transplant recipients [5]. The risk of developing cancer is markedly elevated in this population, with a 2- to 3-fold increase compared to the general population [6][7]. This heightened risk is not uniform across all cancer types. While certain cancers, such as breast, prostate, ovarian, brain, and cervical, do not exhibit a significant increase, others demonstrate a substantial rise. Notably, lung, colon, liver, lymphoma, melanoma, and non-melanoma skin cancers are significantly more prevalent in KT recipients [8].

Furthermore, mortality rates associated with PTM are demonstrably higher in this population compared to the general public [9][10]. Studies report a wide range in PTM incidence (2% to 31%) depending on follow-up duration and specific cancer type, with the risk potentially reaching 50% in long-term follow-up studies [11][7]. The cumulative incidence of post-transplant malignancies can reach alarming levels: 20% after 10 years and 30% after 20 years [12]. Population-based studies conducted in Australia, New Zealand, and Spain have identified a troubling trend: post-transplant malignancy has surpassed cardiovascular disease as the leading cause of death within the first year post-transplant, likely attributable to advancements in preventing cardiovascular mortality [13][14]. A thorough understanding of post-transplant malignancies is crucial for improving patient survival following kidney transplantation. This knowledge can inform the development of effective screening and surveillance protocols for post-transplant malignancies. While previous studies have identified several factors contributing to the increased risk of malignancies in transplant recipients [15], the incidence, mortality, and associated risk factors for PTM exhibit substantial ethnic and geographic heterogeneity. This study aims to address this knowledge gap by investigating the incidence, characteristics, and risk factors specific to PTM in a population of kidney transplant patients.

## Materials and Methods

This research received ethical approval from the Urmia University of Medical Sciences ethics committee (Approval Number: IR.UMSU.HIMAM.REC.1402.030) and was conducted in accordance with the principles outlined in the Declaration of Helsinki.

A retrospective cross-sectional analysis was conducted to evaluate the medical records of all kidney transplant recipients at the Affiliated Hospital of Urmia University of Medical Sciences from January 2000 to December 2020. After meeting the inclusion criteria, by consensus sampling, the patients were assigned into two groups with malignancy and without malignancy in the study.

Demographic and clinical data were retrieved from the hospital’s electronic medical records and paper medical charts. This data encompassed:

* Patient demographics

* Comorbidities (pre-existing medical conditions)

* Details of the transplant procedure

* Past and present cancer history

Immunosuppressive medication regimens

Following transplantation, patients were monitored until the occurrence of one of the following endpoints:

* Diagnosis of a new cancer

* Death from any cause

* Conclusion of the study period

Cancer-related mortality was defined as death directly attributable to a malignancy.

This study included all kidney transplant recipients. Several exclusion criteria were applied:

1. Dual Pancreas-Kidney Transplant: Patients receiving simultaneous pancreas and kidney transplants were excluded.

2. Prior Organ Transplants: Patients who had received any prior organ transplants (liver, pancreas, heart, or lungs) before KT were excluded.

3. Patients with incomplete medical records that prevented comprehensive data collection were excluded from the study.

4. Patients with a documented history of pre-existing malignancy prior to kidney transplantation were excluded. This focus ensured that the study investigated de novo malignancies that developed post-transplant.

The majority of patients underwent induction therapy with either anti-CD25 antibodies or antithymocyte globulin (ATG). Subsequent to induction, all patients transitioned to a standardized maintenance immunosuppression regimen. This regimen typically included a combination of prednisolone (or MycophenolateMofetil for tuberculosis prophylaxis), cyclosporine (Sandimune) or tacrolimus, and azathioprine (or mycophenolatemofetil or everolimus). Prednisolone was additionally administered to treat biopsy-proven T-cell mediated rejection or clinically diagnosed rejection episodes. For patients with steroid-resistant rejection, ATG was used as a second-line therapy. Notably, our center did not perform ABO-incompatible kidney transplants prior to 2020.Patient follow-up continued until the occurrence of one of the following events:

* Diagnosis of a new malignancy

* Death from any cause

* Last documented patient contact

* Study conclusion date (November 30, 2020)

Statistical Analyses

Patients were stratified into malignancy-positive and malignancy-negative groups. Descriptive statistics were presented as percentages for categorical variables and as mean values with standard deviation or median values with interquartile range (IQR, 25%-75%) for continuous variables. To compare the two groups, statistical significance was determined using appropriate tests, including the t-test, Mann-Whitney test, Pearson chi-square test, and Fisher exact test. All analyses were performed using SPSS 21, and a P-value of less than 0.05 was considered statistically significant.

## Results

**Table:** Table1. Demographic Characteristics and Malignancy Incidence of all Patients

Variable	Total (n=4070)	Patients without malignancy (4031)	Patients with malignancy (n=39)	Malignancy incidence per 1000 cases	P-value
Sex					
Male	3042(74.7%)	3018(74.9%)	24(61.54%)	7.89%	0.51
Female	1028(25.3%)	1013(25.1%)	15(33.46%)	14.6%	
Age group (years)					
30>	1747(42.9%)	1739(43.14%)	8(20.51%)	4.6%	
50-30	1708(41.9%)	1695(42.05%)	13(33.33%)	7.6%	<0.001
50<	615(15.2%)	567(14.81%)	18(46.15%)	29.3%	

**Table:** Table2. Clinical Characteristics of Patients with Malignancy

Variables	Variables	N(%)
Transplant number	First Transplant	36(92.3%)
	Retransplant	3(7.7%)
Transplant donor	Related	3(7.7%)
	Unrelated	36(92.3%)
Get ATG	Yes	3(7.7%)
	No	36(92.3%)
	Prednisolone + sandimone + azathioprine	14(35.9%)
	Prednisolone + sandimone + cell sept	14(35.9%)
	Prednisolone + sandimone + cell sept + rapamion	3(7.7%)
	Prednisolone + sandimone + cell sept + tacrolimus	1(2.6%)
Diet therapy	Prednisolone + Cell Sept + Rapamion	1(2.6%)
	Prednisolone + Cell Sept + Tacrolimus	1(2.6%)
	Prednisolone + sandimone	1(2.6%)
	Tuberculosis + tacrolimus	1(2.6%)
	Sandymon + Rapamion	1(2.6%)
	GN	15(38.5%)
	HTN	14(35.9%)
	diabetes	3(7.7%)
Underlying cause of kidney failure	HTN + kidney stones	1(2.6%)
	Neurogenic bladder	1(2.6%)
	Polycystic kidney	1(2.6%)
	kidney infection	1(2.6%)
	kidney stone	1(2.6%)
Receiving immunosuppression before transplantation	No	38(97.4%)
	Yes	1(2.6%)

Chi-square test

A total of 4,070 kidney transplant recipients who underwent transplantation between 2000 and 2020 were included in this retrospective analysis (Table-1). The majority of transplant recipients were male (74.7%, n=3,042), with females comprising the remaining 25.3% (n=1,028). In terms of age distribution, 42.9% (n=1,747) of patients were younger than 30 years old, 41.9% (n=1,708) were aged 30-50 years, and 15.2% (n=615) were older than 50 years old. The mean recipient age at the time of transplantation for those who developed malignancies was 45.64 years (standard deviation ± 15.66). The mean age at the time of malignancy diagnosis was 53.78 years (standard deviation ± 14.24).

Of 4070 transplant recipients, 39 developed PTM (Table-1). The majority of PTM cases occurred in males (61.54%, n=24), with females accounting for the remaining 33.46% (n=15). The distribution of PTM cases across age groups also differed:

* Less than 30 years: 8 patients (20.51%)

* 30-50 years: 13 patients (33.33%)

* Over 50 years: 18 patients (46.15%)

The overall incidence of PTM was 9.6% per 1,000 patients (95% confidence interval: 9.6-13.2). Although the incidence was slightly higher in females (14.6 per 1,000) compared to males (7.89 per 1,000), this difference was not statistically significant (P=0.51). A statistically significant difference (P<0.001) was observed in PTM incidence across age groups. The incidence rates were:

* Less than 30 years: 4.6% per 1000 patients

* 30-50 years: 7.6% per 1000 patients

* Over 50 years: 29.3% per 1000 patients

This data clearly demonstrates a substantial increase in PTM risk with advancing age, with the highest incidence observed in the over-50 age group (Table-1).

The overall incidence of PTM was 9.6% per 1,000 patients (95% confidence interval: 9.6-13.2). Although the incidence was slightly higher in females (14.6 per 1,000) compared to males (7.89 per 1,000), this difference was not statistically significant (P=0.51). A statistically significant difference (P<0.001) was observed in PTM incidence across age groups. The incidence rates were:

* Less than 30 years: 4.6% per 1000 patients

* 30-50 years: 7.6% per 1000 patients

* Over 50 years: 29.3% per 1000 patients

This data clearly demonstrates a substantial increase in PTM risk with advancing age, with the highest incidence observed in the over-50 age group (Table-1). The results showed that living unrelated donors were the primary source of kidneys for first transplants, accounting for 92.3% of cases. A minority of patients (n=3, 7.7%) required the addition of ATG to their primary immunosuppression regimen. The most commonly employed immunosuppressive regimens consisted of prednisolone in combination with either sandimune and azathioprine or sandimune and cellcept, which were utilized in 35.9% of patients. The most common underlying causes of kidney failure were glomerulonephritis (GN) and hypertension (HTN), accounting for 38.5% and 35.9% of cases, respectively. Other causes followed in frequency. A total of 97.4% of patients had not received prior immunosuppression, and only one patient had received pulse cyclophosphamide (Table-2).

## Discussion

Our findings indicated significant differences among studied patients regarding PTM incidence across age groups. A substantial increase was shown in PTM risk with advancing age, with the highest incidence observed in the over-50 age group. Moreover, the results showed that living unrelated donors were the primary source of kidneys for first transplants. The development of malignancies is a well-established complication of organ transplantation [16]. KT is no exception, with a clear association between KT and an increased risk of cancer [17]. This heightened risk remains a primary cause of mortality and morbidity among kidney allograft recipients [17]. Recent data from large kidney transplant registries suggest a possible increase in cancer incidence within this population [18]. The overall reported increase in cancer incidence ranges from 2- to 10-fold compared to the general population, with some studies even reporting a 100-fold increase for specific cancers [19].

This study identified a PTM incidence of 9.58% per 1000 patients, exceeding rates reported in other countries. For instance, studies in Japan documented a PTM incidence of 7.3% [20]. Similarly, research conducted in Western nations among kidney transplant recipients yielded PTM proportions of 4.2% and 7.1% [21]. The observed elevation in overall cancer risk in our study aligns with published data from national population-based studies using standardized incidence ratios (SIRs) for transplant-related malignancies [22]. It is well-established that PTM incidence exhibits geographic variation and depends on the specific type of cancer [22]. Generally, Asian countries, such as Taiwan (3.75%) and Japan (2.78%), tend to report higher standardized incidence ratios (SIRs) compared to Western nations like the United Kingdom (2.4) and the United States (2.1%) [23][24][25][26]. Several factors may contribute to this disparity, such as variations in sample size, study design (hospital-based versus population-based), and the era of transplantation [1].

Our analysis of PTM in kidney transplant recipients revealed a distribution skewed towards males and older individuals. The frequency of PTM cases across age groups also showed a trend towards increased risk with advancing age. These findings regarding sex and age are consistent with several prior studies [1][20][27][28]. However, Jung et al. presented data suggesting no significant sex-based difference in PTM risk, but a higher risk in younger recipients [1]. This discrepancy underscores the potential influence of confounding factors, such as smoking habits, which are more prevalent among males and are known to increase cancer risk. Furthermore, men may be more susceptible to underlying conditions that contribute to kidney failure, and societal pressures associated with busy lifestyles may lead them to neglect preventative health measures [29]. Future research should incorporate data on these potential confounders, such as smoking history and socioeconomic factors, to provide a more comprehensive understanding of the factors influencing PTM risk. This would allow for the development of more targeted preventive strategies for high-risk patient populations.

This study population primarily received kidneys from living unrelated donors (72.3%). While living-related donor kidney transplantation offers advantages, particularly when the donor is a first-generation relative [30], practical challenges often exist. Potential donors may harbor anxieties about long-term health consequences of donation, and patients may be reluctant to accept a kidney from a close relative [31]. Conversely, unrelated living kidney donation, with the exception of altruistic cases, is often driven by financial motivations [32]. Socioeconomic disparities may lead individuals to consider kidney sales as a means to address financial hardship. Despite a potentially higher human leukocyte antigen (HLA) mismatch risk in living-donor compared to deceased-donor kidney transplantation, living-donor transplants generally yield superior outcomes [30][33]. This may contribute to the observed preference for living-donor transplants. Additionally, cultural and religious factors may influence kidney transplantation rates in some regions, with Asian countries potentially in earlier stages of widespread adoption [34]. Future research efforts may benefit from exploring the interplay between socioeconomic factors, cultural beliefs, and donor source selection.

Our study identified that the most common maintenance immunosuppressive regimens employed a combination of prednisolone, cyclosporine (Sandimune), and either azathioprine or mycophenolatemofetil (Cellcept) (each used in 35.9% of patients). This observation aligns with several retrospective studies suggesting a possible link between intensified immunosuppression and a higher incidence of malignancies [35][36]. It is well-established that episodes of acute graft rejection often necessitate increased immunosuppression [37]. This intensification of immunosuppression is demonstrably correlated with a significant rise in PTM rates across various solid organ transplant procedures [38].

The heightened immunosuppression not only increases the risk of PTM development but may also accelerate tumor progression and negatively impact patient survival [38]. While overall immunosuppression plays a critical role in PTM development, the specific immunosuppressive drugs used also have distinct safety profiles [15]. These variations arise from the drugs’ mechanisms of action, targeting specific pathways within the immune response. These pathways may be crucial for immunosurveillance, antiviral defense, or, in some cases, may even possess direct oncogenic potential [15]. Therefore, it is essential to consider the potential influence of individual immunosuppressive medications within the context of PTM risk. Future research efforts should explore this area further to optimize immunosuppressive regimens that effectively balance graft protection with reduced PTM risk.

The most common underlying causes of kidney failure requiring transplantation in our study population were GN and HTN, accounting for 38.5% and 35.9% of cases, respectively. These findings align with a recent study reporting hypertension as a significant contributor to kidney failure, at 35.6% [39]. Moreover, a systematic review and meta-analysis documented a high prevalence (17.8%, 95% CI: 13.0-23.3%) of chronic kidney disease (CKD) among hypertensive patients in sub-Saharan Africa [40]. Differentiating hypertensive nephropathy from other causes of kidney failure can be complex. Hypertension can accelerate the progression of pre-existing renal insufficiency, while kidney disease itself can induce secondary hypertension [41]. Glomerular diseases are a well-documented cause of kidney failure, but their specific characterization can be limited due to lower rates of renal biopsy procedures [42]. This limitation is further supported by a study among hemodialysis patients, where hypertension and chronic glomerulonephritis were identified as the leading causes of kidney failure [43]. These findings emphasize the importance of early diagnosis and management of both hypertension and glomerular diseases to potentially prevent or delay the progression to kidney failure and the need for transplantation.

Limitation of Study

This study is subject to several limitations inherent to its retrospective design. While data collection was performed by trained investigators to minimize errors and ensure completeness, the retrospective nature precludes the ability to establish causality for observed associations. Furthermore, our database lacked information on established risk factors for post-transplant malignancies, such as cigarette smoking, alcohol consumption, family history of cancer, and particularly analgesic abuse, which has been linked to an increased risk of malignancies. Additionally, the analysis may be limited by the dynamic nature of immunosuppressive regimens in kidney transplant recipients. Changes in immunosuppressive medications over time were not captured in detail, potentially hindering our ability to fully assess the impact of immunosuppression on post-transplant malignancy development. Future prospective studies with comprehensive data collection on established risk factors and detailed immunosuppressive regimens are warranted to provide a more robust understanding of the factors influencing post-transplant malignancy risk in this patient population.

## Conclusion

This study identified a higher PTM incidence compared to data from other countries. This observation warrants further investigation to elucidate the underlying mechanisms contributing to this disparity. Analyses revealed that male sex, older recipient age, and a history of pre-existing medical conditions were significant predictors of post-transplant malignancy development. Additionally, the study population primarily received kidneys from living unrelated donors. Future research efforts should explore the potential influence of various factors, including potential socioeconomic disparities and cultural attitudes towards deceased and related organ donation, on donor source selection in this population. Furthermore, public health initiatives promoting organ donation registries and encouraging families of deceased individuals to consider organ donation are crucial to expand the pool of available organs and improve transplant outcomes.

## Acknowledgments

The authors would like to express their gratitude to the clinical research development unit of Imam Khomeini Hospital, Urmia University of Medical Sciences, for English editing.

## Conflict of Interest

The authors have no competing interests to declare that are relevant to the content of this article.
